# Organizational journeys toward strong cultures of sustainability: a qualitative inquiry

**DOI:** 10.3389/fpsyg.2025.1508818

**Published:** 2025-02-13

**Authors:** Manuel Riemer, Joel Marcus, Benjamin Kai Reimer-Watts

**Affiliations:** ^1^Department of Psychology, Wilfrid Laurier University, Waterloo, ON, Canada; ^2^Viessmann Centre for Engagement and Research in Sustainability (VERiS), Wilfrid Laurier University, Waterloo, ON, Canada; ^3^School of Administrative Studies, York University, Toronto, ON, Canada

**Keywords:** culture of sustainability, organizational culture, sustainability, qualitative study, stages of development, organizational leaders, systems-thinking

## Abstract

**Introduction:**

There is widespread belief that organizational culture plays a crucial role in transitioning organizations for sustainability, but we currently lack understanding of how supportive cultures develop. The goal of this study is to empirically investigate how a culture of sustainability (COS) develops within a varied sample of real-world organizations.

**Methods:**

A qualitative cross-sectional design was utilized in this study using 14 semi-structured qualitative interviews with leaders of organizations perceived as having a strong COS or being on a good path toward that. The interviews explored how the leaders from various organizations experienced the development process of a COS from the initial emergence to the time of the interview. The qualitative data were analyzed using template analysis combined with applying a team-based approach to open coding.

**Results:**

The results indicate that while COS development is not a direct, clear, or linear process, there are several common factors that descriptively capture the process of COS formation. The analysis revealed four general stages of COS development (emergence; visibility and engagement; institutionalization and system alignment; ingrained and habitualized practice) and three key contextual moderators (organizational characteristics; external stakeholders/societal culture; business case).

**Discussion:**

This study makes an important contribution to the limited empirical literature on the development of organizational culture over time. Understanding key factors, relationships between factors, and COS stages can help leaders establish realistic expectations and strategies for developing and strengthening COS within their organizations.

## Introduction

Organizational behavior scholars have called attention to organizational culture in relation to sustainability issues for more than two decades (e.g., Harris and Crane, 2002). The question of whether organizational culture is ‘fit for purpose’ to help facilitate the sustainability transition is of particular interest, especially in business organizations whose social and environmental impacts weigh heavily on prospects for a more sustainable future (Hawken, 1993). Cultural factors are central to sustainability concerns because human activities (the causal factor underlying our currently unsustainable societal systems) are largely driven, maintained, and conditioned by mental models and cultural norms, standards, and expectations (Dreyer et al., 2021; Kania et al., 2018; Packalén, 2010). In a substantive sense, the challenge of sustainability is a cultural challenge. To meet this challenge, there is a need to reorient values, beliefs, worldviews, objectives, human practices, and more directly, the individual and collective activities that are compromising the viability of sustaining life on this planet (Macy and Johnstone, 2022; Caesar et al., 2024).

Despite this understanding, scholarly efforts pertaining to organizational culture of sustainability (COS) have been relatively limited and sporadic, and we have much to learn about the role COS plays in organizational life and how such cultures develop. In this paper, we take a holistic view of sustainability as encompassing environmental, social, and economic systems (Marcus et al., 2010), and understand COS as “shared values, symbols, rituals, and practices grounded in sustainability principles leading to individual and societal choices that promote environmental protection, social justice, and well-being, and a supportive economy” (Dreyer et al., 2021). The purpose of our qualitative study is to better understand how leaders in organizations with a perceived strong COS and/or a clear commitment to advancing sustainability objectives understand the COS development process within their organizations.

A number of complex systems models aiming to explain COS processes have been proposed in recent years (Assoratgoon and Kantabutra, 2023; Dreyer et al., 2021; Norton et al., 2015), and somewhat ahead of actual research findings, numerous applied works have also been issued, intended to guide management practice and help managers create a strong COS within their organizations (Bertels et al., 2010; Galpin et al., 2015; Howard-Grenville and Gapp, 2022; Leleux and van der Kaaij, 2019). While these efforts highlight the perceived importance of COS, existing models and frameworks have had little direct empirical testing and are almost exclusively drawn deductively from the literature rather than built from direct observation of what is happening within firms. Consequently, our understanding of what leading organizations do to develop and/or strengthen a culture of sustainability remains limited (cf. Howard-Grenville and Bertels, 2011) and the extent to which culture can be shifted to enact more sustainable outcomes remains mainly an open question.

A number of additional questions remain unanswered: How does COS develop in real-world organizations? What process and practices are firms following and with what outcomes? Are there commonalities and/or relevant differences in why, when, and how a COS develops, and what it means for the firms, employees, and broader stakeholder groups? Overall, what are the dominant features, characteristics, elements, processes of COS development, and are there observable patterns across organizational type, size, and industry?

Our current study seeks to better understand the COS development process through the lived experience of leaders in organizations with a demonstrably strong COS and/or a clear commitment to advancing sustainability objectives. Sustainability management is an applied discipline whose concerns are rooted in highly consequential real-world phenomena and effects. Thus, it is especially important to connect theory to practice when considering how to best shift organizational cultures toward sustainability. Observing what is happening within firms is central to knowledge generation, sharing, and learning (i.e., praxis) for organizational and societal change.

As compared to the majority of conceptual development in the area that has employed a form of narrative synthesis based on existing literature (Galpin et al., 2015), we set out to first identify a sample population of organizations with a known strong COS or clear commitment to advancing sustainability objectives, and then examine what these organizations have done that might contribute to the development and achievement of a strong COS within their workplace. For this purpose, we conducted in-depth interviews with fifteen leaders from fourteen small to medium-sized organizations to assess the unique COS development process within each organization. Our approach is informed by systems thinking; that is, we are especially interested in identifying key factors and relationships between them that bear upon COS development in a holistic, integrated manner (Dreyer et al., 2021).

Our findings complement and advance previous work and indicate that: (a) COS development is not a clear, linear process, and can be rather messy, chaotic, and iterative (Galpin et al., 2015) (b) There is no simple, step-by-step process that will with any great degree of certainty lead to a strong COS; (c) However, there are several common identifiable factors that impact COS development across organizations; (d) Further, there are various stages of COS development that can be defined, despite being somewhat fuzzy.

Understanding the factors, relationships between factors, and COS stages can help leaders to position their organizations within complex realities and to focus on the most relevant aspects to advance COS development from where they are currently. Although we have aimed for organizational diversity within our sample set (e.g., age, industry, size, type) we note at the outset that our sample population of mostly small to medium-sized enterprises, drawn from only a few geographic regions within Canada does not begin to capture the diversity of organizations within Canada, let alone globally. Hence, our findings will exhibit range restriction and should be considered an early attempt to understand COS development processes within organizations.

In the next section, we review the literature on COS and related works to provide background and contextualization for the current study. We then describe the methods of our study including design, sampling, procedures, and analytical approach. Following this, we report the key findings of our qualitative analyses and a process model of COS development derived from these findings. We conclude with a discussion of key insights and implications for theory, practice, and future research.

## Background and literature review

Scholarly interest in organizational culture as it relates to sustainability has been wide ranging, spanning numerous disciplines, methodologies, approaches, and purposes. This is to be expected because it reflects the interdisciplinary and multidimensional nature of the underlying phenomena and variables of interest. Although this research area is characterized by a lack of systematic development, the relevant literature can be loosely grouped into three categories that reflect the primary focus of a given work. These include: (a) conceptual/theoretical, (b) empirical, and (c) practitioner-oriented contributions.

Conceptual and theoretical papers have typically sought to define the construct domain for sustainability culture within organizations (e.g., Aggarwal and Agarwala, 2021; Harris and Crane, 2002; Howard-Grenville and Bertels, 2011) or to develop relational models centered on culture as the focal construct. Several dynamic systems models attempting to conceptually delineate this phenomenological space have been presented in recent years (Assoratgoon and Kantabutra, 2023; Dreyer et al., 2021; Galpin et al., 2015; Kantabutra, 2021; Ketprapakorn and Kantabutra, 2022; Norton et al., 2015), reflecting a literature still developing, still in flux, and not fully coherent.

On the empirical front, researchers have assessed the direct, mediating, and moderating effects of culture in relation to such variables as pro-environmental employee behavior (Khan and Terason, 2022; Norton et al., 2014) marketing strategy and firm performance (Fraj et al., 2011), firm environmental performance (Afum et al., 2020; Magsi et al., 2018) and quality improvement and sustainability performance (Fok et al., 2022). This body of findings largely confirms that sustainability culture does have significant and meaningful effects within organizations, though it must be noted that research studies are relatively scattered and small in number.

Consistent with this orientation, but somewhat surprising given the limited empirical work, numerous practitioner guides have been issued outlining practices, frameworks and roadmaps by which managers might develop a culture of sustainability within their organizations (Bertels et al., 2010; Galpin et al., 2015; Howard-Grenville and Gapp, 2022; Leleux and van der Kaaij, 2019). It is especially notable that these managerial prescriptions and purported best practices are not, as of yet, grounded in studies of how COS develops in real-world organizations, but rather informed by deductive theorizing from literature reviews and/or standard, general management principles and frameworks. Nevertheless, they serve to highlight the strong belief many scholars share regarding the importance of sustainability culture.

Across the varied efforts to date is a clear, widespread expectation that organizational culture plays a key role in either facilitating or hindering progress toward sustainability, and some amount of evidence – which we would regard as preliminary – to support that belief. Overall, the related literature shows considerable breadth but relatively little depth in terms of systematic, concentrated or cohesive research efforts and findings. Given the complex nature of the underlying phenomena, we do not believe that achieving a fully unified, singular view is possible or even desirable, and see value in bringing multiple perspectives and approaches to address questions of culture and sustainability. However, this does present challenges for clear scholarly communication and cumulative knowledge development, and makes it difficult to encapsulate the literature succinctly. Subsequently, we do not intend to present a comprehensive review of COS-related literature here, which is beyond the scope of this paper and is covered in a recent contribution by Assoratgoon and Kantabutra (2023). Rather, because our objective is to ‘look inside’ and better understand how COS develops within real organizations, our review simply aims to situate and justify the current study within the broad contours of existing work. As a first step, it is necessary to address the conceptual meaning of COS (cf. Locke, 2012).

### Key concepts and definitions

Our adoption of ‘culture of sustainability’ as the focal construct in this research follows a developing stream of research (Bertels et al., 2010; Dreyer et al., 2021; Leleux and van der Kaaij, 2019) but must be understood in terms of its component elements and situated with respect to closely related concepts. It is notably a compound concept, combining ‘culture’ and ‘sustainability.’ Organizational culture and sustainability, in turn, are both multifaceted and contested concepts, each reflecting the complex nature of the phenomena they address. As a meta-concept, ‘culture of sustainability’ has even greater potential for a broad range of interpretations and confusion. We note a plethora of closely related and/or identical concepts in the literature, including sustainability organizational culture (Assoratgoon and Kantabutra, 2023), green organizational culture (Aggarwal and Agarwala, 2021; Harris and Crane, 2002; Tahir et al., 2019), pro-environmental organizational culture and climate (Norton et al., 2015; Piwowar-Sulej, 2020), green climate (Flagstad et al., 2021) and green psychological climate (Norton et al., 2017).

Among this diverse terminology, two substantive distinctions stand out (i.e., culture vs. climate and sustainability vs. environmental), both of which reflect the conceptual breadth of the respective construct under consideration. In both these cases, we adopt the broader, more encompassing framing.

#### Organizational culture vs. climate

Organizational culture and organizational climate are closely related concepts that are commonly used interchangeably. Despite attempts to clarify their distinctiveness (Denison and Mishra, 1995; Schneider et al., 2014; Norton et al., 2015), conceptual conflation remains the norm and in many cases the practical difference is obscure at best (see Flagstad et al., 2021). When distinctions are made, culture is generally seen as being deeper and more encompassing – rooted in basic values and assumptions that manifest in a variety of forms in the life of an organization – whereas organizational climate is conceived as shared perceptions of the work environment (Flagstad et al., 2021; Schneider et al., 2014). Perhaps because of this, the body of work examining organizational culture is more established, both in the general management literature and with respect to sustainability issues. Culture also has the additional benefit of being more purely a social construct, as compared to climate, which is a weather analog drawn from the physical sciences. We subsequently retain culture – instead of climate – as our focal construct in this paper.

#### Sustainability vs. environmental

A number of scholars in this line of inquiry have looked at organizational culture more narrowly in relation to environmental issues and activities (Howard-Grenville, 2006) and/or pro-environmental (or “green”) organizational culture/climate. Environmental issues have also been captured under the banner of “social initiatives” (Howard-Grenville and Hoffman, 2003). We follow those using the broader term of sustainability, both for its current prominence in scholarly and practitioner circles, and because it holistically encompasses environmental, social, and economic factors, which helps to highlight systemic interdependencies that are essential to understanding aggregate outcomes and impacts of interest.

#### Culture of sustainability (COS)

Numerous definitions of COS have been presented in the literature (Ketprapakorn and Kantabutra, 2022) and we are cautious of further muddying the waters by attempting yet another formulation here. We note that, despite differences in framing and emphasis, there seems to be general agreement that COS refers to shared values, symbols/artifacts, and rituals/practices that relate holistically to objectives and outcomes across the environmental, social, and economic domains of sustainability (Assoratgoon and Kantabutra, 2023; Bertels et al., 2010; Dreyer et al., 2021). These definitions generally build from Schein’s (1986) foundational work that conceptualizes organizational culture as a complex construct with both hidden (e.g., commonly shared understandings, worldviews, beliefs, and values) and visible aspects (i.e., culture manifests in a variety of more tangible ways in the life of an organization such as via symbols and common practices) (Baumgartner, 2009). Though not explicit within Schein’s schema, an additional manifestation of import is the language, stories, and myths that circulate in the communication environment (Pettigrew, 1979). Together these more visible elements form the normative context which condition the behavior of organizational members, reinforce underlying values, assumptions and beliefs, and ultimately act to perpetuate and stabilize organizational culture over time.

Our understanding of COS is similar to others in terms of its comprehensive form and constituent elements (Linnenluecke et al., 2009), but is further informed by an embedded perspective that highlights hierarchical dependencies between systems and their relative importance (Marcus et al., 2010). Thus, we prioritize social and environmental objectives over organizational-level outcomes and diverge somewhat from those who seek win-win-win outcomes or to simply ‘balance’ objectives (Assoratgoon and Kantabutra, 2023; Bertels et al., 2010; Galpin et al., 2015). We see organizational goals and organizational sustainability as secondary to broader societal and environmental sustainability because there is no possibility of the former without the latter.

### Key insights from existing literature

Despite the somewhat messy and developing state of the literature, there are some prominent characteristics, themes, and findings that can be highlighted and which inform our work. These include: (a) an orientation toward *multidimensionality and systems thinking*, (b) a prominent *practitioner focus*, and (c) the identification of *key* var*iables and processes* including the interplay between leaders and employees, personal values, and the broader societal context within which firms operate.

#### Multidimensionality and systems thinking

There appears to be near universal agreement on the importance of applying systems thinking to understand and promote COS in organizations. Systems-thinking “is a set of synergistic analytic skills used to improve and understand the system as a whole, by identifying underlying systemic structures and understanding how different system parts work together to produce specific practices and devise modifications to them in order to achieve desired goals and objectives” (Dreyer et al., 2021, p. 6). With a deeper understanding of the systems and its dynamics, leverage points for intervening in the system and creating transformative change can be identified (Meadows, 1997, 2009).

The multidimensional nature of cultural greening, for example, emerged as a key finding from Harris and Crane’s (2002) leader interviews, which is one of the first studies to explicitly examine organizational culture in relation to environmental issues. Their qualitative analysis revealed three dimensions of green organizational culture (depth, degree, and diffusion) along with seven factors that account for variance across those dimensions. Harris and Crane’s (2002) model of green culture appears in rather static form, and numerous authors have since developed causal models that aim to capture the key factors and relationships at play. Galpin et al. (2015) present a linear theoretical model that encapsulates multiple organizational processes, with sustainability culture mediating firm strategy and sustainability performance at both the organizational and individual levels. Similarly, Assoratgoon and Kantabutra (2023) develop a model of COS (encompassing sustainability vision, values, leadership, and practices) that proposes direct effects on corporate sustainability, and Kantabutra (2021) extends the linear path further, suggesting COS ultimately impacts brand equity.

More ambitious models portray dynamic interactions and feedback loops among a broad set of complex organizational-system components. Such dynamic models have been proposed by Norton et al. (2015) to explain how pro-environmental cultures and climates emerge, and by Dreyer et al. (2021) to illustrate a theory of change for COS development. Ketprapakorn and Kantabutra (2022) likewise propose a holistic, systems model with sustainability culture at the heart of organizational processes. It is notable that, besides Harris and Crane (2002), these models have all been derived deductively and have undergone little, if any, empirical testing. This represents a significant gap between COS theory and observed reality.

To inform the analytical approach of our study, we drew primarily from the theoretical model proposed by Dreyer et al. (2021). These authors combined the dynamic system model by Foster-Fishman et al. (2007) with applications from a review of relevant literature on COS in organizations and organizational change. While the model was developed to explain relationships between the social and physical aspects of green office buildings, leader-employee dynamics and contextual impacts on COS are also recognized and especially relevant to our current study. Their emphasis on engaging organizational actors aligns with our interest in studying COS development as dynamic process over time. Dreyer et al. (2021) highlight five key principles in the development of organizational COS that we paid attention to during our interviews and analysis: systems-oriented, long-term developmental, strategic, comprehensive, and participatory.

#### Practitioner focus

Conceptual analyses far surpass empirical research in this literature, with narrative synthesis being the dominant mode of conceptual and theoretical development (see Galpin et al., 2015). As noted earlier, the fact that most models have been deductively (not empirically) generated has not limited prescriptions for practice and, overall, the literature shows a strong practitioner orientation. Bertels et al. (2010), for instance, performed a systematic review of the literature to develop their guideline for embedding sustainability in organizational culture. They develop a multi-dimensional circumplex model and assessment tool encompassing two primary poles (fulfillment vs. innovation and informal vs. formal) and 59 distinct practices. They suggest that “organizations should draw from all four quadrants in their efforts to embed sustainability…we speculate that a balanced approach is required” (p. 18).

Galpin et al. (2015) similarly draw from “practitioner and empirical literature from various disciplines” to “develop a comprehensive model that serves as a blueprint for leaders attempting to create a culture of sustainability within their organizations” (p. 3). Their ‘culture of sustainability organizational model’ incorporates firm mission, values, goals, strategy, human resource practices, and firm-level outcomes. They indicate that, despite appearing as a series of linear steps, the cultural development process is iterative. Similarly, in their work to establish a culture of sustainability in green buildings, Dreyer et al. (2021) develop a particularly comprehensive and dynamic theoretical model considering physical building attributes, building citizens, influence and engagement dynamics, and sustainability outcomes. Supplemental to their theoretical contribution is a separate practitioner guide.

While these models and guidelines appear quite reasonable and have a considerable degree of face validity, in the absence of testing and fieldwork it remains unclear whether the proposed approaches are truly effective and which of the many factors considered may be of particular importance. It is also not clear whether practicing managers in firms with strong COS are paying much attention to these guidelines, or whether they are achieving COS results by some other means.

#### Key variables and processes

The plethora of variables brought under consideration in this literature is far too vast to fully consider here. Given this wide scope, one is tempted to conclude that in the realm of organizational culture “everything matters” – which while in some measure true is of little practical merit. With consideration of the shortcomings of the literature noted above, there do appear to be some key factors and processes that cut across the literature that can be identified. These are largely captured in a recent contribution by Flagstad et al. (2021) whose qualitative study to understand the process of establishing ‘green climate’ in small, entrepreneurial firms in Norway precedes and closely parallels our efforts here. It is perhaps the only existing study of climate/culture formation processes prior to our own.

Consistent with preceding conceptual work (Baumgartner, 2009; Galpin et al., 2015; Schneider et al., 2014) Flagstad and colleagues find *leadership* to be of particular importance in establishing green climate with *employees* playing a key role in climate maintenance, which they describe as a mutual-influence process. It is not surprising that founders in small firms have an outsized impact on organizational culture and climate, but this is also observed in much larger corporations as well such as Patagonia (founder Yvonne Chouinard) and Interface Carpet (founder Ray Anderson). The observed interplay between *top-down and bottom-up mechanisms* appears to also confirm prior theoretical work (Dreyer et al., 2021; Schneider et al., 2014).

Flagstad et al. (2021) also found that *environmental practices* are a main focus in firms with strong ‘green climate’, and that these practices are driven less by extrinsic factors (e.g., regulation, stakeholder expectations) and more by intrinsic motivations and *personal values*. In fact, these practices are used to spread organizational values to the broader community. It also appears that leaders in these firms were often unable to articulate their values, which suggests that they exist at the level of underlying assumption as proposed by Schein’s (1986) original model. In nearly all the works surveyed here, basic individual and organizational values are seen to be the bedrock of organizational culture.

Contrary to what has been widely hypothesized (Galpin et al., 2015; Ketprapakorn and Kantabutra, 2022), Flagstad et al. do not find evidence that overarching strategy, vision, and missions have much impact on green climate development. One possible explanation for this is that strategizing and visioning may be less common in small entrepreneurial firms under resource constraints, as compared to larger more established firms. However, the authors suggest that the strong commitment to the environment within the broader Norwegian society may also play a role.

To this latter point, Flagstad et al.’s systems model and findings highlight how the broader macrosystem acts as a conditioning factor on green organizational climate. Such a macrosystem can have a significant and dampening effect, potentially slowing organizational culture change. That organizational culture change is slow and difficult, and the observed lack of progress in developing strong cultures of sustainability in organizations despite the worsening global climate change trajectory has been a repeated theme in the literature from the beginning (Bertels et al., 2010; Harris and Crane, 2002). Yet, this seemingly glacial pace is perhaps not surprising given that organizational existence likely requires that organizational culture not stray too far from the dominant societal culture within which it is embedded. Nonetheless, we might facilitate faster organizational transitions toward sustainability by better understanding the process by which COS develops, especially within organizations demonstrating a relatively stronger COS.

Although our research was undertaken without knowledge of Flagstad et al.’s study and findings, it clearly represents a close parallel effort to better understand organizational culture development processes for sustainability. Our independently developed study focused on the following guiding research question: How do leaders in organizations with a perceived strong COS and/or a clear commitment to advancing sustainability objectives understand the COS development process within their organization?

## Methods

For this study we used a qualitative cross-sectional design using semi-structured qualitative interviews with leaders of organizations perceived as having a strong COS or being on a good path toward that. Our research is informed by a constructivist research paradigm, which emphasizes that people create their own reality through their experience. Our goal was to understand how the leaders from various organizations experienced the development process of a COS from the initial emergence to the time of the interview.

### Sampling and recruitment

For our sample population, we identified organizations with a strong COS and/or a clear commitment to advancing sustainability using multiple approaches. First, participants were identified based on a pre-existing dataset from a national study that focused on COS in organizations. Second, leaders from two intermediary organizations (one national and the other regional) that support organizations in becoming more environmentally sustainable (e.g., by setting targets, providing information about relevant greenhouse gas reduction opportunities, and supporting employee engagement strategies) were recruited. We asked these leaders to identify organizations that they perceive as fitting our criteria above (i.e., having a strong COS or being on a good path toward that). Third, we recruited three organizations already known to the research team as being sustainability leaders. Finally, during the interview we asked participants to nominate other organizations that they deemed relevant to the study. In our sampling strategy, our objective was not to focus on one type of industry but to explore similarities across a diverse sample of organizations (i.e., maximum variation sampling).

### Participants

For each organization we reached out either to an executive leader with knowledge about COS development in their organization (mostly for smaller organizations) or the individual overseeing sustainability initiatives. This led to a diverse sample of leaders from 14 organizations from across Canada, with a majority located in the broader locale of the regional intermediary organization and the lead researchers in Southwestern Ontario. The sample included a broad variety of both non-profit organizations (e.g., a large university, a music festival) and commercial companies (e.g., a large insurer, a small manufacturer, a brewery co-op, a large building management company) ranging from between 10 and 34,000 employees, with the majority in the range of 10–50. The participants included founders and CEOs of smaller companies, execute directors of organizations, a worker co-owner of a cooperative organization, and higher-level sustainability managers in larger companies and organizations. Despite our intent to recruit primarily high-COS organizations, one leader noted that sustainability was relatively new for their organization and that they did not consider the organization to have a strong COS at the time of the interview. Overall, while the sample is characterized by relatively strong COS, organizations demonstrated different stages of development with respect to their COS journey.

### Procedures

Each leader was contacted via email by the principal investigator. If the leader agreed to participate, they received a follow-up email from the project manager with an invitation to schedule the interview and a request to complete the consent form. Consent forms were collected prior to conducting the interview. Interviews were conducted online via Zoom at a time convenient to the participant. Within one organization, two leaders asked to complete the interview together, resulting in a final sample of interviews from 14 organizations. The first two interviews were conducted by the lead author and one or both of the PhD-level research assistants (RAs). The remaining interviews were conducted by one of the RAs. Each semi-structured interview was scheduled for 1 hour and lasted between 41 and 79 min with an average length of 61 min. All procedures were approved by the Research Ethics Review Board of the principal investigator.

### Data collection method

The interview participants were provided with a definition of COS similar to the one provided above and derived from Dreyer et al. (2021) (“*We define a COS as characterized by shared values, symbols, rituals, and practices grounded in sustainability principles leading to individual and societal choices that promote environmental protection, social justice, and well-being, and a supportive economy*.”). Participants were also informed that we were interested to learn about factors that have contributed to the development of the COS within their organization, and the direction they see their organization headed in with respect to sustainability. Interview questions targeted the COS development process, factors contributing to the development of COS, the role of leadership, their aspirations for continuing to maintain and potentially strengthen a COS, and their recommendations for other leaders trying to promote COS. Each interview was recorded and transcribed to text, and the interviewers wrote research memos with reflections immediately following the interviews.

### Analysis

**Figure 1:**
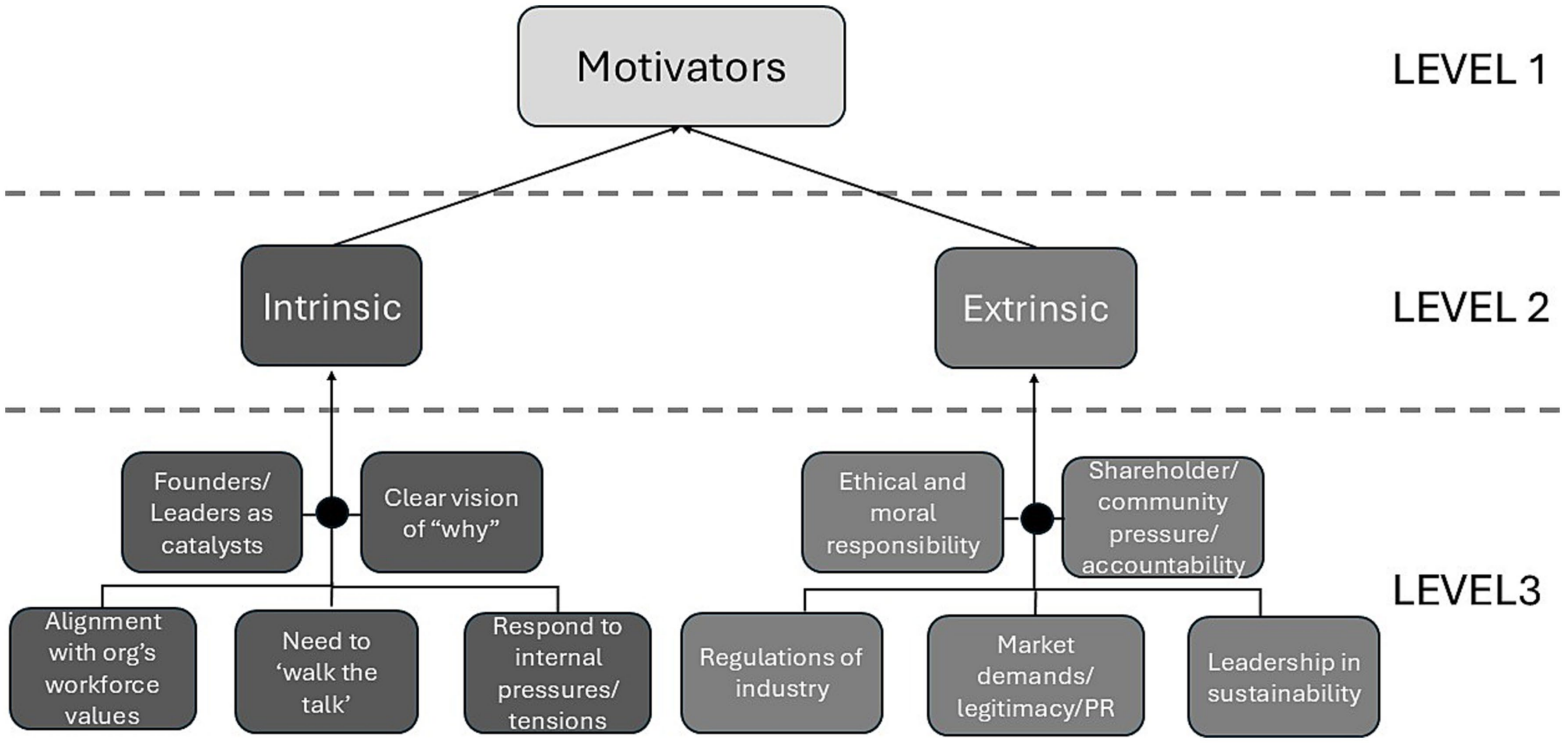
Example of hierarchical coding structure.

We analyzed the data within the tradition of thematic analysis (Braun and Clarke, 2006), which is well aligned with the constructivist research paradigm. Specifically, we used template analysis, which is a type of thematic analysis that “emphasizes the use of hierarchical coding but balances a relatively high degree of structure in the process of analysing textual data with the flexibility to adapt it to the needs of a particular study” (King et al., 2012 in Brooks et al., 2015, p. 203). For this analytic approach, Brooks and colleagues propose six general steps: (1) familiarize yourself with the data, (2) carry out preliminary coding of the data, (3) organize emerging themes into meaningful clusters, (4) define an initial coding template, (5) apply the initial template to further data and revise as necessary, and (6) finalize template and apply it to all data. We combined the template analysis with a team-based approach to open coding that emphasizes intercoder consensus as described by Cascio et al. (2019). Coding of the interview transcripts was done using the cloud-based analysis software Dedoose, which is suitable for team-based analysis of qualitative data. Similarly to Cascio et al., we present two examples of our coding and analysis process below to demonstrate our analytical process (see Figure 1; Table 1) rather than include the full record of our coding process, which is too extensive to include in this paper.

**Table 1:** Example of consensus process in team-based open coding approach.

	Researcher 1		Researcher 2	
Verbatim quotations from transcript	Open codes^a^	Axial codes^b^	Open code	Axial codes
They have to have a clear vision for their organization, with core values that’s supported around this culture of sustainability…	Have a clear vision	Clear vision for “why”	Clear vision for their organization with core values	Clear vision for “why”
As long as even a few members care, it’s going to get brought up, it’s going to become part of operations and planning… our key concern is the interest of the members…	If members care it will get brought up	Alignment with workforce values/priorities	Concerned with interest of the members	Alignment with workforce values/priorities
This is where the world is headed… And it’s also the right thing to do…	The right thing to do	Ethical and moral responsibilities	The right thing to do	Ethical and moral responsibilities
People will come to us because they buy into what we are doing… That’s what we are trying to build… it’s a sustainable business model…	It’s a more sustainable business model	Supports more sustainable business model	People will come to us as they buy into what we are doing	Buying into what we are doing

^aThese are in vivo codes that draw heavily from the language used by participants themselves.^

^bThese codes group open codes into common themes around shared “axes” of meaning.^

The preliminary coding (Level 3 codes) of four interviews was conducted by two pairs (comprised of one principal researcher and one RA) using the team-based consensus coding process so that each interview was coded twice. For this initial coding, in-vivo codes were used. That is, the coders developed open codes that stayed relatively close to the language of the interviewee. Each pair would then meet to discuss their in-vivo codes and collaboratively develop axial codes based on these. An example of this process is shown in Table 1. In cases of disagreement, these were further discussed and resolved among the whole team. As seen in the final example showing some early disagreement in Table 1, the team settled on the axial code “*Supports more sustainable business model.*” For each axial code, the team then developed a definition that would be consistently applied through the coding of remaining interviews, as well as selected one or more exemplary quotes that illustrate the code well. For the example above, the definition is “*COS development encourages/enables organization(s) to operate under a more sustainable business model.*” All four researchers would then meet and discuss the codes being generated, using the online whiteboard program Miro to visualize and organize codes into clusters (i.e., Level 1 and 2 codes) collaboratively. An example of this can be seen in Figure 1 for the Level 1 code “*Motivators*.”

**Figure 2:**
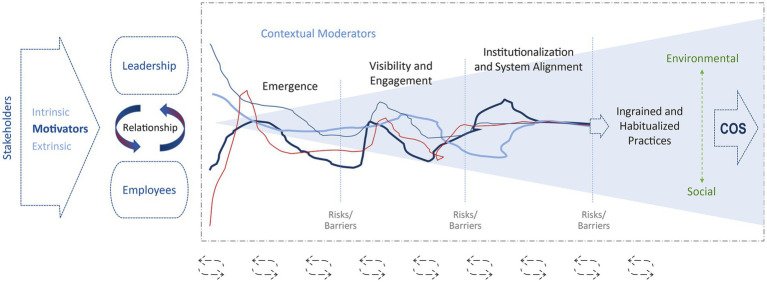
Thematic model: organizational cultures of sustainability development.

A preliminary codebook was then established and applied to three more interviews, following which the group met again to discuss any changes to the codebook. At this stage, special attention was given to the reduction of codes through merging of similar codes. Following this process, the two RAs coded the remaining interviews. In the end, there were 268 Level 3 (child) codes, 41 Level 2 (parent) codes, and 13 Level 1 (grandparent) codes in the codebook. The team used various analytical matrices to do cross-interview thematic analysis based on the coded interviews (Miles et al., 2019). Over several iterations the team generated the key themes that are presented in the Results section below, and developed multiple concept maps to visualize the relationships between higher-level codes and themes. The goal here was to identify the most relevant themes related to the research question and to understand their relationships in the context of a developing COS within organizations. For a cluster to be considered a theme, it needed to be represented within at least four interviews. However, for categories that are clustered within a theme, such as the various examples of organizational moderators, this was not an expectation as the goal here was to show the breadth of types within this theme. The end-result of this process is represented in Figure 2 in the Results section.

Following the standards of writing up qualitative research using thematic analysis, we then selected two to three exemplary quotes to illustrate each theme. These quotes, and the context within which they were to be used (i.e., excerpts of the manuscript), were then shared with interviewees to ensure their agreement with our interpretations. While some participants requested small edits of their quotes, none disagreed substantively with our interpretations, providing confidence in our final results. For further validation, we then presented our results and the inductively generated model in Figure 2 to various experts in sustainability in organizations for a critical review before finalizing this manuscript.

## Results

Despite the prominence of prescriptive guides in the literature, our interviews revealed that COS development varied significantly across organizations and is not a clear, linear process, but instead an often complex, messy, and iterative process that takes considerable time, as expressed by this organizational leader:

Unless there’s an external trigger point, like a Coronavirus, or, you know, the George Floyd protests, you do not normally have rapid change in culture. Because there’s not normally a rapid realization that you are doing something completely wrong. And so, you have to build on it. And it really is a thing that you change over time. [PRO4].

The initiation of COS was also seen to have different starting points and to be influenced by various contextual factors, with different weight and influence across organizations. Despite these differences across organizations, we were able to discern four general qualitative stages in the development process that are described further below. The stages are comparable in nature to the stages Piaget famously observed in the cognitive development of a child (Bovet, 1976) in that they (a) are qualitatively distinct but blurry in the transition from one stage to the next; (b) progressive in that the earlier stage prepares for the following stages (with the possibility of regression into earlier stages); and (c) can manifest themselves in different ways and with different timelines across organizations. This finding of the somewhat blurry and varied ways in which COS develops within organizations is represented by the nonlinear colored lines at the center of the model shown in Figure 2.

The key stages and potential factors influencing each stage are captured in Figure 2 and Table 2. It is important to emphasize that this is not a deterministic or predictive model but rather one that is process-oriented, capturing potential emergent factors at four different stages in the development process as well as initial drivers and supporting contextual factors. We believe that the value of this model lies in its ability to clarify expectations at each stage along the COS journey so that emergent opportunities can be better noticed and seized. As noted by one of our participants from a production company: “If you are not ready, when the opportunity strikes, you are not going to… you got to seize every opportunity.” [PRO4].

**Table 2:** Contextual moderators.

Organizational characteristics	External stakeholders/societal culture	Business case
Origin / history Industry type Organization size Materials and resource use (e.g., building, supply chains) Organizational culture and climate Leadership style	Influence of external stakeholders Influence of community/societal culture	Perceived ROI for sustainability Risk mitigation or reduced risk exposure Employee attraction and retention Reduced operating costs

### Overview of COS developmental model

Below we present the key themes and their relationships as they were generated through our analytical process described above logically by following the flow of the model in Figure 2, which also represents the flow of COS development as we discerned it from our interviews. The COS development model seen in Figure 2 and Table 2 illustrates both the overall complexity, organic nature, and non-linearity of the COS development process (thus the nonlinear lines shown in the model). This model also provides insight into some general stages of COS development, identifiable factors that may influence COS development, and how leaders, champions, and other change facilitators can better position their organization within this complex space.

Despite the horizontal flow of the model, it is intended to show an organic evolution of COS that may initially be sparked by either *leadership* or *employees* based on various intrinsic and extrinsic *motivators* (often driven by internal or external *stakeholders*), and then progress in a variety of different ways (visualized by non-linear colored lines in the model). Over time, these efforts must converge in order for broader organizational system alignment and institutionalization to occur, which may progress at different speeds for instance depending on the relationship between leadership and employees, and other factors. The development of COS, despite being somewhat fuzzy, can be usefully divided into four stages based on our participants’ experiences: (1) *Emergence*; (2) *Visibility and Engagement*; (3) *Institutionalization and System Alignment*; and (4) *Ingrained and Habitual Practices*.

As noted above, our participating organizations exhibited different stages in this journey at the time of their interviews. That is, some were relatively early in the journey (not self-described as having a strong COS) whereas others had a mature COS, developed and solidified over many years of implementing and refining sustainability practices. Also, while all participants emphasized the *environmental* aspect of sustainability within their COS, the degree to which they discussed the *social* aspects of this culture was more variable. The model also shows *feedback cycles* as represented by circular arrows at the bottom, which visually reinforce the likelihood for non-linear progression through the stages, as well as the possibility of regression to previous stages. At each stage there can be *barriers* to progress (e.g., lack of leadership commitment) and risks of either regressing to earlier stages or dissolution and leaving the COS journey altogether (although this was not present in our sample).

What seems consistent is that organizational COS is an ongoing journey and not a distinct end state, even though there are clearly organizations with much stronger COS compared to the majority. In the following sections, we elaborate each of the major components of the COS development journey.

### Theme 1: drivers and motivators

Participants described a variety of intrinsic and extrinsic motivations and drivers for beginning their COS journey. Most were influenced by both internal and external stakeholders. *Intrinsic motivators* are those that emerge from within the organization either directly from the leadership or driven by employees. For example, relevant personal values and experiences of leaders were a strong driving force, especially for smaller organizations where the leader(s) hold more direct power. In sharing his COS origin story, one participant noted that he grew increasingly frustrated with the lack of investment in and support for social and environmental sustainability among the multi-national consulting companies he worked for. He described preferring work that “is prevention based, which is very good economically, environmentally, socially, but it’s not really good for the consultant company itself” [CON2] because it diminishes future work opportunities. Not satisfied with the mainstream consulting approach, this participant decided to start up his own company and embed a COS into the fabric of the organization, attracting employees aligned with that vision. Another leader described the birth of his child as a pivotal moment. Still others described alignment of sustainability with the overall company’s values and priorities and a need “to walk the talk.” As one participant noted:

It starts with kind of our key motivation and what we try and do as a company, which is to “bring life to communities,” our tagline. And so, everything we do is all about building community. And whether that’s better, more sustainable developments, quality homes, or the charitable work we do in the region – it’s all in the end about building community. [HOM]

Leaders also cited employee-led initiatives and pressure from employees to become more sustainable as a motivator to act on COS.

*Extrinsic motivators* include pressure and encouragement from various external stakeholders, such as shareholders, consumers, and the local communities. Participants described the need to “not fall behind” industry peers, to demonstrate leadership in the industry, and to comply with industry regulations. One leader, for example, talked about the reputational risk of not acting on sustainability:

You know, what does this mean reputationally for our company? What does this mean, for a number of different areas on our risk assessment if we aren’t successful in leading the way in a number of sustainability initiatives? [EVE2]

A worker-owner of a brewing co-op and a leader of a music festival, on the other hand, both described the importance of being embedded within the local community and how that influenced their focus on sustainability. In the case of the music festival:

I think sustainability has meant a lot to the organization. […] Right from the start, it was founded and organized around the sort of kitchen tables and campfires of musicians, but people who were also environmental activists. And the relationship to the land has always been a constitutive part of our festival, identity, and value system. It has attracted the participation of a lot of volunteers and people who had an interest in environmentalism. From the start, there was an urge to do things differently. [FES]

### Theme 2: stages in the COS development process

#### Stage one: emergence

The first stage in the COS development process can best be described as *Emergence*. At this stage, the initial development is sparked by early ideas and enthusiasm by passionate individuals or subgroups within the organization for development of a shift in culture toward a stronger emphasis on sustainability. As one participant noted:

It starts with the passion and purpose. Without the passion and purpose, without the fire, without that spark, you cannot ignite and create this movement and shift in sustainability culture. [CON1]

Often this effort is not coordinated and may only appear in certain parts of the organization, especially when they are larger. As mentioned above, the initial spark can be a result of various motivations and drivers and can come from leadership, employees, or other stakeholders. One sustainability leader described how the COS movement started within their organization:

So, I was the lonely nut out there. But [a colleague] saw that I was doing something great and she was my first follower, if you will – but I wasn’t a leader until she agreed to follow. Now, my style of leadership is “okay, come on, let us go. I’m no longer the lonely nut, we are two lonely nuts so we can lead. We’ll find other lonely nuts, and we’ll all figure this shit out together.” [EVE1]

For some organizations, this spark occurred very early on in their history, even at their foundational stage, while for others it happened much later or took longer to spread across the organization. Having internal champions with high levels of motivation for change and who are willing to swim against the stream initially was seen by several of our participants as key to this stage. A sustainability lead for a larger company expressed it this way:

At [company] our commitment to sustainability has blossomed from grassroots efforts. Key team members, especially one of our key architects, fueled by [their] passion for sustainability, began sharing this vision across the team. This grassroots enthusiasm was met with strong support from senior management [fellow architect], [CEO], and [civil engineer], who wielded significant influence within the organization. [ARC]

Importantly, these initial steps may not be easily identifiable as the beginning of a COS, as there may not be a conscious intention to develop a COS but rather an unconscious outflow of basic values, worldviews, and ideals that organically evolve into the development of COS. While internal organizational champions often play a role in instigating the initial “spark,” for COS to develop further it requires a range of supports, including efforts to broaden engagement with and visibility of the emergent culture across an organization. This marks a transition into the second stage.

#### Stage two: visibility and engagement

For the initial spark to turn into a broader movement and culture shift across the organization, the second stage of *Visibility and Engagement* is critical. Securing leadership support and commitment was seen as important especially for those organizations for which the COS was sparked by employee champions or somewhere at the periphery of the organization. As one participant framed their need for added leadership support, “We had the spokes throughout the business, but we did not necessarily have the hub” [INS]. Another participant explained how they advocated with their board of directors to invest in sustainability with a different approach than they might use in employee-level conversations to include “a little bit more emphasis on the risks that are associated with not doing this type of work.” [EVE2]

In contrast, in organizations where the sustainability shift was initiated among the leadership, the leaders talked about the importance of engaging employees. As one participant put it: “our [organizational leaders] cannot make culture, they can just give permission, in a sense for things to happen” [UNI1]. Employee and stakeholder engagement can be done through informal and formal engagement and clear communications. This includes communication of the importance of focusing on sustainability through sustainability-focused value and vision statements, setting of sustainability goals and reporting on progress toward these, education, and by forming green teams of voluntary internal champions. One participant described this stage as follows:

From the passion and purpose, it moves to the vision, creating the vision for the company, with a set of core values that are focused on sustainability. We developed our vision and developed our set of core values that the company lives by each day. We took that, and we inspired and motivated our employees. These core values are from top management (the decision makers), but it’s the employees that put those words into action by supporting and sharing in the sustainability culture of the company. [CON1]

This quote also speaks to the need to create visibility for the culture shift that was mentioned by several participants, such as linking sustainability to core values and creating a shared vision for sustainability. As discussed in the introduction, values are at the core of organizational culture, but unless these are communicated through symbols and artifacts and translated into shared social practices, the values will have limited impact on the development of COS. Sustainability symbols can take a variety of forms such as a poster to encourage waste reduction/diversion, solar panels, or a communication of the organization’s commitment to sustainability on their website – among many others. The leader of the music festival, for example, shared how they created visibility for sustainability by putting up “little billboards, showing people how to create a solar water heater.” Other visibility was created through electricity-generating bikes and by showing patrons how they “can cycle in order to charge your cell phone [or] to make yourself a chocolate drink.”

The visibility and engagement stage is an important step toward institutionalization and deeper integration of sustainability into the organizational culture more broadly, which characterizes Stage Three. However, it is also easy to get stuck in this second stage, especially when the key driving forces for promoting COS are primarily external factors (e.g., customer pressure or competition with other companies) rather than a deeper internal motivation. Also, there is a risk that, because of their relative ease and low cost, visible sustainability signs and activities may be over-prioritized at the cost of achieving a deeper, more substantive state of COS within the firm. All of these reasons suggest a need to eventually evolve from this stage into deeper institutionalization and system alignment with sustainability.

### Stage three: institutionalization and system alignment

In this stage the integration of sustainability becomes more in-depth and the organization itself starts to change. A commitment to sustainability becomes part of a shared organizational identity and the purpose of the organization may also change to be better aligned with that identity. One participant described this process as follows:

The alignment of our core values, from both the senior management level and the grassroots, has been instrumental. Prioritizing sustainability throughout the organization allowed our sustainable initiatives to take root and flourish. This widespread support across all levels of the firm has been the cornerstone of our enduring commitment to sustainability. [ARC]

In Stage Three, sustainability starts to become strategically integrated and systematically structured throughout the organization, involving such things as setting environmental targets, standardizing sustainability process and practices, and the designation of formal sustainability roles. For example, one leader shared that they:

Put a committee in place that’s accountable for, every year, trying to measure what [our] footprint is, and then come up with an initiative that will bring down that footprint. I mean, that was a game changer for us. [FES]

Another participant talked about how sustainability considerations can become self-sustaining over time:

The staff remembered past initiatives and simultaneously came up with new project ideas to implement and helped to ensure that past projects stayed active and ran year after year. This way projects like motor upgrades or incentive plans for staff produced self-sustaining benefits like employee retention and cost savings. Knowing that we have literally over 100 sustainability projects that benefit the company continuously in the background, all while we focus on looking after customers, is a huge relief to us as business owners. [MET]

There was a shared sentiment that this type of institutionalization (or normalization) of sustainability and system alignment was important in the COS development to avoid being stuck in a stage where sustainability communications surpass day-to-day actions. As one participant noted: “If culture change means anything, it means that it’s not just a few policies at the top, it’s actually the organization as a whole.” [UNI1] Later in the interview the same person emphasized:

In the end, I think what we have to do is normalize sustainability. As long as sustainability is the change, we are failing. As long as we have to change to be sustainable, we are failing. Partly because we snap. There’s a persistence problem in all these programs, people behave differently for a little while, and then they snap back to the [previous] norm. We have to change the norm.

Hence, in this stage important processes, structures, and habits are established that create a strong base for the next phase in which sustainability is now normalized and deeply embedded in every aspect of the organization.

#### Stage four: ingrained and habitualized practices

This fourth stage can be considered the most mature form of COS, with sustainability now embedded throughout the organization and represented by a close alignment between underlying values, organizational practices (e.g., purchasing, marketing, employee hiring and management, client engagement, product and supply chain, and day-to-day practices of employees), and the physical aspects of the organization (e.g., buildings, technology). Engaging in sustainability practices and decision-making becomes more automatic and habitualized at this stage. Whereas sustainability is a largely deliberate and conscious activity in previous stages, in Stage Four it takes shape as an underlying assumption governing all organizational life:

I find that if you are doing that in every facet of what you are doing, it just becomes a habit and it just becomes subconscious. And it just becomes an instinct that when you approach something, make a decision, answer something – sustainability is just always there. [PRO1]

The environmental, social, and financial dimensions of sustainability are also well-integrated at this stage, as shared by the leader of an environmental consulting company:

We address all aspects of sustainability, from the people to the planet and profit, that’s another way of looking at it. In all our decisions and all our business activities. So, it’s not just a service that we provide, we live by it, and it is ingrained in our daily actions. It’s something that we have embraced in all our operations… This is where this whole culture of sustainability can really thrive. [CON1]

It’s important to note that only a few of the organizations in our sample represented this fourth and final stage of our model at the time that we interviewed them. Those that did tended to be smaller, had sustainability as a core value embedded from the outset, and viewed sustainability holistically (i.e., as being more than only environmental). This stage is also not to be seen as an end state at which an organization can become complacent, as maintaining a strong COS is an ongoing journey. However, the normalization and habitualization of sustainability throughout an organization that our participants described makes it less of a change effort at this stage.

### Theme 3: challenges, barriers, feedback cycles, and the need for ongoing maintenance

Our participants described the development of a COS as an emergent and non-linear process going through iterative feedback cycles, which is depicted by the circular arrows at the bottom of the model in Figure 2. Developing a COS is a challenging, lengthy process that one participant described as “climbing mount sustainability” in reference to Ray Anderson from Interface. Another noted:

It’s a process. And I think that’s what I’m afraid of, is that it’s a bandwagon and a lot of people are going to want to just get to the end – not realizing it is a process, it’s going to take time. [PRO1]

There are opportunities along the journey for steps to strengthen a COS – for instance by learning from and amplifying “what works” – as well as risks of stagnation and regressing backwards. Participants noted, for example, that they encountered a strategic challenge in coping with certain trade-offs associated with COS development, such as the ability to balance short-term returns with long-term planning so as to warrant continued investment. It can also be challenging to balance the trade-offs and priorities across the economic, environmental, and social dimensions of sustainability. Being able to measure and demonstrate progress holistically to maintain buy-in from key stakeholders is especially important, as experienced by this leader of a larger organization:

Because sustainability is often so qualitative and it’s not easily quantified, in many ways we have needed to find ways to quantify sustainability initiatives and measure them in a way where it demonstrates how we are becoming more successful as a business. [EVE2]

At each stage, including Stage Four, there is danger of falling back into previous stages when external factors (e.g., societal priorities, market conditions) and internal circumstances change (e.g., turn-over of champions, loss of institutional knowledge, new leadership). The societal understanding of sustainability and its scope also constantly changes, such as the increasing emphasis on social considerations (e.g., equity, diversity, and inclusion) in the context of environmental, social, and corporate governance (ESG) frameworks. Our participants shared that it is important that a COS adapts with these changes, recognizing a need to always consider how to maintain and evolve the existing COS and not rest on previous accomplishments. Clearly, a COS is not a self-perpetuating end-state but instead requires ongoing commitment and maintenance, as expressed well in this quote:

I do not think anything can be self-perpetuating. I think the leader’s role is to make sure there are other leaders who are feeding into the vision and the implementation of that vision, and that they feel passionate about it and committed… I always try to pick staff who feel passionate about things because I know that they are people who are going to be ‘on fire’ and will learn something. [FES]

### Theme 4: contextual moderators

As part of the COS development process, interviewees discussed a number of moderating factors that affect the efficacy of efforts supporting COS development. These moderators were contextual to their organization, its business model, its people, and the communities within which it is embedded. Contextual factors moderating the COS development process exist both within and beyond an organization’s operational boundaries. We found that these contextual factors could be sorted into three overarching categories: (1) Organizational characteristics, (2) External stakeholders and societal culture, and (3) Business case (see Table 2).

There are a variety of *organizational characteristics* that we found influenced the speed and scope of COS development among these organizations, including: origin and history; organizational type; industry type; organizational size, materials and resources use (e.g., buildings, supply chains); general organizational culture and climate; and leadership style.

A subset of interviewees, for example, explained that their organization had a clear vision of and value for sustainability since the organization’s inception. This influenced both the speed and scope of development of COS, for example, by attracting a workforce comprised of individuals whose personal values aligned with the organization’s focus on sustainability and were personally committed to advancing it. Leaders made specific reference to both social (e.g., community-building; investing in communities) and environmental (e.g., environmental stewardship) values already embedded in their general organizational culture as enhancing employee and other stakeholder buy-in. In contrast, those leaders of organizations that did not already have these pre-existing values reported more challenges in getting COS development off the ground.

Organizations with worker-ownership models and components (e.g., cooperatives, non-profits) had a comparatively easier time with COS development. Participants listed numerous reasons for this positive relationship including that worker-ownership models are built around their members and are often community-based and accountable to the local community rather than a distant shareholder. Their democratic governance is also empowering to the employees, which fosters engagement in organizational initiatives, including those focused on sustainability.

Small organizational size was perceived as positively affecting COS development, especially during the *Visibility and Engagement* phase, in part because of the visibility of leaders’ role-modeling behaviors. Leaders of these organizations reported greater ease in engaging their entire workforce simultaneously while the resources and time required to facilitate desired behavior changes was reduced.

*External stakeholders and overall societal culture* were also considered influencing factors. For example, we previously described the impact of being closely connected to local communities on COS development (i.e., external stakeholders). Beyond this, leaders of profit-oriented companies talked about the influence of societal culture more broadly through market demands and reputational pressures. One leader, for example, shared that:

The minute the financial markets become interested in something, it makes it that much easier to achieve. And so, we now see the federal government wanting to have all kinds of green funds and infrastructure. To me, it’s like we have been striking the match for 25 years and something finally caught fire for those of us trying to change the world. [PRO1]

Leaders also referenced society’s growing sustainability awareness in relation to workforce development, indicating that people entering the workforce hold sustainability as a personal value, and in turn want to work for sustainable organizations. However, given that fostering COS is a long-term and uncertain process, a lack of shareholder support was perceived as impeding the development process and requiring additional efforts to further sustainability agendas.

It seems self-evident that having a good *business case* for investing in sustainability helps with the development of a COS as it generates support among leaders and other financially-driven stakeholders. One leader, for instance, talked about how seeing the positive financial pay-off of emissions reduction investments spurred further action on sustainability:

Then we realized, hey, this really has a real financial impact – I can tell you that we did not expect meaningful benefits to our bottom line, only a reduction in our greenhouse gas emissions. At this point we started tackling and calculating our emissions. In fact, between 2006 and 2008 we implemented 37 sustainability initiatives which cut our carbon footprint by 50% even though we doubled our building size from 11,400 sq. ft. to 26,000 sq. ft. The data shows that the initiatives had an average ROI of over 200% and payback was less than 6 months.

The experience of seeing that sustainability can have an incredible financial impact was inspiring to our team to the point that we continued coming up with new initiatives. By 2023 we had implemented at least 116 projects and more than doubled our profit as a result, and for the last seven years the company achieved carbon neutral status. [MET]

Another leader talked about how earlier champions made the business case for investing in COS development with their corporate leadership:

They said “OK corporate level, you have to get with this” because … even from a customer perspective, there was, “Are we going to start missing out on deals because we cannot show that [company] as a business we are taking this very seriously, and that it is part of our culture?” [INS]

### Theme 5: perceived value and impacts of COS

Overall, our participants perceived a COS as important and valuable for their organization. One leader, for example, believed that a COS is important because of its impact on people’s behavior:

From issues [such] as reducing energy and water consumption, achieving a sustainable culture will require people to change their behaviors. You can invest in all the smart technology you want, but unless you educate and can change people’s behavior, you’ll not achieve the true potential of sustainability. [CON1]

A similar sentiment was expressed in this statement on the benefit of COS:

I think any business that has people, which is all of them, and uses some resources, which is all of them, would benefit from some sort of sustainability consideration, planning, and practice. [BRE]

Another participant talked about the positive impact of investing in COS on the overall organizational climate and engagement of employees:

We need to figure out how to engage and activate people’s interest in reducing emissions. I can add this amazing anecdote as proof of the benefits of accomplishing this goal… In 2021 I sold my company and the new owners did a culture assessment in early 2022 comparing our division against 4 other companies they owned. Our division scored highest in every category. This was definitive proof that our focus on sustainability had tangible benefits beyond our bottom line. While we had grown our sales to record levels, we had simultaneously built a far more positive work environment, achieved low staff turnover, doubled our profits, plus achieved broad community engagement. We cannot stress enough that all the above success started with an initial focus on energy reduction which expanded over time to include procedures, policies, training, employee incentives and more. [MET]

This relates to another participant’s perceptions on how COS can help increase organizational resilience, including that “having a culture of sustainability made us resilient through this [COVID-19] pandemic” [CON1]. This leader also emphasized the importance of having some structure and vision for developing a COS for support, such as via a COS roadmap or guide:

When you can create a roadmap or a guide, that will help steer them in the right direction. Think of it as “I know where I want to get to, but if I do not have a map, it’s going to take me a heck of a lot longer to get there than if I have a map.” [CON1]

## Discussion

In this study we examine the role of organizational culture in facilitating a transition toward sustainability. We were especially interested in empirically investigating how organizational cultures of sustainability develop within organizations and what factors may influence the development. For this purpose, we sampled extreme cases (Patton, 2002) of organizations who were considered “top of the class” regarding their COS development and interviewed their leaders about their experiences. We sought to learn from those who were relatively successful in developing a COS or on a good path in that direction. While this provides us with valuable insights about how COS develops, this study does not provide much insight into the barriers faced by organizations who fail to develop a meaningful COS, as for example Harris and Crane (2002) were able to provide through their research.

As compared to the study by Flagstad et al. (2021), we did not limit ourselves to one organizational or industry type but instead recruited a diverse sample of organizations. This allowed us to identify commonalities across types and sectors and to draw conclusions that are more easily transferable to a broader range of organizations and allows for a richer assessment of this complex phenomenon. However, because we limited our sample to organizational leaders, it is reasonable to expect that employee perspectives on COS development will provide additional and potentially contradictory insights. For example, Flagstad et al. (2021) suggest that “while leaders might have a bird’s eye-view of organizational greening, employees tend to have a hands-on approach to practice and procedures” (p. 21). Readers should keep these considerations in mind when interpreting our findings.

Our participants’ insights align with Harris and Crane (2002) regarding the fundamental importance of culture in moving organizations forward on sustainability. Consistent with the common understanding of COS (Assoratgoon and Kantabutra, 2023; Bertels et al., 2010; Dreyer et al., 2021) interviewees described the need for aligning organizational values with sustainability goals, policies, and practices, for visibility of sustainability initiatives, and broad engagement of both employees and leadership.

Our empirical findings also align with previous theoretical accounts of COS as a complex dynamic process that develops within a systems context (Dreyer et al., 2021; Harré et al., 2022; Ketprapakorn and Kantabutra, 2022; Norton et al., 2015). Our participants described COS development as an organic process that takes time and patience. The insight shared by one of the leaders in this study that one has to be part of the system and ready to ‘make a move’ when the opportunity emerges, agrees particularly well with Harré et al.’s (2022) people-focused systems approach to sustainability based on their research with a school in Auckland, New Zealand. Drawing from systems thinking, Harré and colleagues describe an organization, such as a school, as being composed of complex systems in which people interact in ways that generate emergent properties that are difficult to predict and prescribe. A key aspect of trying to create change in complex dynamic systems is that one has to manage *in* complexity rather than attempting the management *of* complexity, which requires working with the dynamics that unfold (Cooke-Davies et al., 2007). The practical implication of this is that COS development cannot be prescribed through a simple step-by-step process. Each organization must navigate its own process based on its own unique context, goals and history.

However, our findings indicate that organizations commonly pass through four general stage categories that descriptively capture COS development. Understanding these stages and what is most relevant at different points along the development journey can help establish realistic expectations and focussed attention on the most relevant factors for further strengthening a COS. For example, it may be premature to set an ambitious carbon reduction target in the early stages before the culture has matured enough to fully and holistically support progress toward that target. In such cases the target may quickly become performative.

Finally, while environmental sustainability dominated our conversations with organizational leaders, we did hear interesting examples of social sustainability efforts as well. For example, we learned that the owner of a small engineering company is providing employees the opportunity to co-own a significant part of the company, even though they do not operate formally as a co-op. In addition, we learned that several organizations provide employees with opportunities to be engaged in the community or that the organization itself is involved in community efforts. One example is a real estate management company that allows victims of domestic violence to quickly get out of their rental agreements prematurely. While the emphasis on the environmental dimension of sustainability is now common in the corporate world, the social dimension is gaining traction with the increasing adoption of environment, social, and governance (ESG) frameworks (Tsang et al., 2023).

## Limitations

Throughout this paper, we have pointed to some limitations related to the scope and methodological choices we made. For example, although we have aimed for organizational diversity within our sample set (e.g., age, industry, size, type) our sample population of mostly small to medium-sized enterprises, drawn from only a few geographic regions within Canada does not begin to capture the diversity of organizations within Canada, let alone globally. Hence, our findings will exhibit range restriction and should be considered an early attempt to understand COS development processes within organizations. Also, we only interviewed leaders (not employees) within the sampled organizations, which may limit the insights regarding the COS development process in these organizations. Finally, we asked participants to recollect their memories regarding the COS development process within their organizations. This recollection could be biased based on how they see their COS currently and the loss of specific memories. However, given the relatively limited previous empirical investigation of this issue, we believe our grounded research both confirms aspects of existing theoretical models and provides novel insights into the COS development process and relevant conditioning factors.

## Conclusion and future research

There is little doubt that companies and other organizations play a critical role in responding to the climate crisis, and it is encouraging that there is an increased interest in developing organizational COS. However, there are currently very few examples of organizations that have actually developed a strong COS. Hence, we sought to explore these phenomena in real-world organizations exhibiting a relatively advanced COS. Our study complements and extends Flagstad et al.’s (2021) recent findings by engaging a varied sample of organizations, specifically selected for having a strong COS. Our empirical model inductively derived from the interview data provides a guide to future researchers who are interested in studying the COS development process, and will help scholars position organizations within specific developmental stages. This would facilitative examination of the contextual factors most critical at different stages. Which factors matter most at which stage may also differ by the type of organization (for-profit vs. non-profit, small vs. large, etc.), and the combination of stages and contextual factors that we have identified provide future researchers with a blueprint for studying these relationships more systematically. Compared to existing practitioner guides on COS development, our findings and model do not provide a prescriptive step-by-step guide, but rather provide insights into the process of COS development across a varied organizational sample that can hopefully inspire further engagement. This more flexible approach also provides the practitioner with the opportunity to develop a COS development strategy that is targeted to their specific context but grounded in the experience of other sustainability leaders.

Given our discussion above, opportunities for future research include (a) observing the development of COS in organizations concurrently rather than relying on the historical account of leaders; (b) trying to actively foster COS in the context of an action-oriented case study; (c) including employee perspectives on COS development in their organization; and (d) obtaining a larger sample of organizations with a sufficient number of various organizational types (e.g., size and industry) to allow for a systematic comparison across types.

## Data Availability

The datasets presented in this article are not readily available because the sensitive qualitative data from the interviews are not publicly available due to institutional restrictions on the sharing of data that includes potentially identifying information and the confidentiality agreement with the interview participants. Requests to access the datasets should be directed to Wilfrid Laurier University Research Ethics board; reb@wlu.ca.

## References

[ref1] Afum E. Agyabeng-Mensah Y. Owusu J. A. (2020). Translating environmental management practices into improved environmental performance via green organizational culture: insight from Ghanaian manufacturing SMEs. J. Supply Chain Manage. Syst. 9, 31–49.

[ref2] Aggarwal P. Agarwala T. (2021). Green organizational culture: an exploration of dimensions. Glob. Bus. Rev. doi: 10.1177/09721509211049890 [Epub ahead of print].

[ref3] Assoratgoon W. Kantabutra S. (2023). Toward a sustainability organizational culture model. J. Clean. Prod. 400:136666. doi: 10.1016/j.jclepro.2023.136666

[ref4] Baumgartner R. J. (2009). Organizational culture and leadership: preconditions for the development of a sustainable corporation. Sustain. Dev. 17, 102–113. doi: 10.1002/sd.405

[ref5] Bertels S. Papania L. Papania D. (2010) Embedding sustainability in organizational culture: a systematic review of the body of knowledge. Network for Business Sustainability. Available at: https://embeddingproject.org/pub/resources/EP-Embedding-Sustainability-in-Organizational-Culture.pdf (Accessed: 21 February 2023).

[ref6] Bovet M. (1976). “Piaget’s theory of cognitive development and individual differences” in Piaget and his school: A reader in Developmental Psychology. eds. Inhelder B. Chipman H. H. Zwingmann C. (Berlin, Heidelberg: Springer), 269–279.

[ref7] Braun V. Clarke V. (2006). Using thematic analysis in psychology. Qual. Res. Psychol. 3, 77–101. doi: 10.1191/1478088706qp063oa

[ref8] Brooks J. McCluskey S. Turley E. King N. (2015). The utility of template analysis in qualitative psychology research. Qual. Res. Psychol. 12, 202–222. doi: 10.1080/14780887.2014.955224, PMID: 27499705 PMC4960514

[ref9] Caesar A. Özdemir O. Weber C. Wermter S. (2024). Enabling action Crossmodality for a Pretrained large language model. Nat. Lang. Process. J. 7:100072. doi: 10.1016/j.nlp.2024.100072, PMID: 39898364

[ref10] Cascio M. A. Lee E. Vaudrin N. Freedman D. A. (2019). A team-based approach to open coding: considerations for creating Intercoder consensus. Field Methods 31, 116–130. doi: 10.1177/1525822X19838237

[ref11] Cooke-Davies T. Cicmil S. Crawford L. Richardson K. (2007). We’re not in Kansas anymore, Toto: mapping the strange landscape of complexity theory, and its relationship to Project Management. Proj. Manag. J. 38, 50–61. doi: 10.1177/875697280703800206

[ref12] Denison D. R. Mishra A. K. (1995). Toward a theory of organizational culture and effectiveness. Organ. Sci. 6, 204–223. doi: 10.1287/orsc.6.2.204, PMID: 19642375

[ref13] Dreyer B. C. Riemer M. Spadafore B. Marcus J. Fernandes D. Taylor A.. (2021). Fostering Cultures of Sustainability in a Multi-Unit Office Building: A Theory of Change. Front Psychol., 12:624311. doi: 10.3389/fpsyg.2021.624311 34040558 PMC8142860

[ref14] Flagstad I. Johnsen S. Rydstedt L. (2021). The Process of Establishing a Green Climate: Face-To-Face Interaction between Leaders and Employees in the Microsystem. JVBL, 14, 25–57. doi: 10.22543/0733.141.1343

[ref15] Fok L. Zee S. Morgan Y.-C. T. (2022). Green practices and sustainability performance: the exploratory links of organizational culture and quality improvement practices. J. Manuf. Technol. Manag. 33, 913–933. doi: 10.1108/JMTM-11-2021-0439

[ref16] Foster-Fishman P. G. Nowell B. Yang H. (2007). Putting the system back into systems’ change: a framework for understanding and changing organizational and community systems. Am. J. Community Psychol. 39, 197–215. doi: 10.1007/s10464-007-9109-0, PMID: 17510791

[ref17] Fraj E. Martínez E. Matute J. (2011). Green marketing strategy and the Firm’s performance: the moderating role of environmental culture. J. Strateg. Mark. 19, 339–355. doi: 10.1080/0965254X.2011.581382

[ref18] Galpin T. Whitttington J. L. Bell G. (2015). Is your sustainability strategy sustainable? Creating a culture of sustainability. Corp. Gov. 15, 1–17. doi: 10.1108/CG-01-2013-0004

[ref19] Harré N. Blythe C. McLean L. Khan S. (2022). A people-focused systems approach to sustainability. Am. J. Community Psychol. 69, 114–133. doi: 10.1002/ajcp.12550, PMID: 34460117 PMC9292166

[ref20] Harris L. C. Crane A. (2002). The greening of organizational culture: management views on the depth, degree and diffusion of change. J. Organ. Chang. Manag. 15, 214–234. doi: 10.1108/09534810210429273

[ref21] Hawken P. (1993). The ecology of commerce: A declaration of sustainability. New York: HarperCollins, Harper Business.

[ref22] Howard-Grenville J. (2006). Inside the “black box”: how organizational culture and subcultures inform interpretations and actions on environmental issues. Organ. Environ. 19, 46–73. doi: 10.1177/1086026605285739

[ref23] Howard-Grenville J. Bertels S. (2011). “Organizational culture and environmental action” in The Oxford handbook of business and the natural environment. eds. Bansal P. Hoffman A. J.. Oxford Academic. (Oxford University Press), 194–210.

[ref25] Howard-Grenville J. Gapp T. (2022). Organizational culture for sustainability, handbook on the business of sustainability. Cheltenham, Glos, UK: Edward Elgar Publishing, 138–151.

[ref26] Howard-Grenville J. Hoffman A. J. (2003). The importance of cultural framing to the success of social initiatives in business. Acad. Manag. Perspect. 17, 70–84. doi: 10.5465/ame.2003.10025199

[ref27] Kania J. Kramer M. Senge P. (2018). The water of systems change: Harvard Business School. Available at: https://www.fsg.org/wp-content/uploads/2021/08/The-Water-of-Systems-Change_rc.pdf

[ref28] Kantabutra S. (2021). Exploring relationships among sustainability organizational culture components at a leading Asian industrial conglomerate. Sustain. For. 13:1733. doi: 10.3390/su13041733

[ref29] Ketprapakorn N. Kantabutra S. (2022). Toward an organizational theory of sustainability culture. Sustain. Produc. Consump. 32, 638–654. doi: 10.1016/j.spc.2022.05.020

[ref30] Khan M. S. Terason S. (2022). Encouraging pro-environmental behavior in university employees: an approach toward environmental sustainability as moderated by green organizational culture. J. Community Psychol. 50, 1454–1469. doi: 10.1002/jcop.22726, PMID: 34626493

[ref31] King N. Symon G. Cassell C. (2012). Doing Template Analysis.’, in Qualitative Organizational Research: Core Methods and Current Challenges. 1st Edn. London: SAGE Publications, Limited, 426–450.

[ref32] Leleux B. van der Kaaij J. (2019) ‘Encouraging a culture of sustainability’, in Leleux B. Kaaij J. van der (eds) Winning sustainability strategies: Finding purpose, driving innovation and executing change. Cham: Springer International Publishing, pp. 153–170.

[ref33] Linnenluecke M. K. Russell S. V. Griffiths A. (2009). Subcultures and sustainability practices: the impact on understanding corporate sustainability. Bus. Strateg. Environ. 18, 432–452. doi: 10.1002/bse.609

[ref34] Locke E. A. (2012). Construct validity vs. concept validity. Hum. Resour. Manag. Rev. 22, 146–148. doi: 10.1016/j.hrmr.2011.11.008

[ref35] Macy J. Johnstone C. (2022). Active Hope (revised): How to face the mess We’re in with unexpected resilience and creative power. Novato, CA, US: New World Library.

[ref36] Magsi H. B. Ong T. S. Ho J. A. Hassan A. F. S. (2018). Organizational culture and environmental performance. Sustainability, 10, 2690. doi: 10.3390/su10082690

[ref37] Marcus J. Kurucz E. C. Colbert B. A. (2010). Conceptions of the business-society-nature Interface: implications for management scholarship. Bus. Soc. 49, 402–438. doi: 10.1177/0007650310368827

[ref38] Meadows D. (1997). Places to Intervene in a System. Whole Earth, 91:78–84. Available at: https://donellameadows.org/wp-content/userfiles/Leverage_Points.pdf

[ref39] Meadows D. (2009) in Thinking in systems: A primer. ed. Wright D.. 1st ed (London: Routledge).

[ref40] Miles M. B. Huberman A. M. Saldaña J. (2019). Qualitative data analysis: A methods sourcebook. Fourth Edn. Los Angeles: Sage.

[ref41] Norton T. A. Zacher H. Ashkanasy N. M. (2014). Organisational sustainability policies and employee green behaviour: the mediating role of work climate perceptions. J. Environ. Psychol. 38, 49–54. doi: 10.1016/j.jenvp.2013.12.008

[ref42] Norton Thomas A. Zacher Hannes Ashkanasy Neal M.. (2015). “Pro-Environmental Organizational Culture and Climate.” in The psychology of green organizations, edited by Barling J. Robertson J. L.. (New York: Oxford University Press).

[ref43] Norton T. A. Zacher H. Parker S. L. Ashkanasy N. M. (2017). Bridging the gap between green behavioral intentions and employee green behavior: the role of green psychological climate: employee green behavior. J. Organ. Behav. 38, 996–1015. doi: 10.1002/job.2178

[ref44] Packalén S. (2010). Culture and sustainability. Corp. Soc. Responsib. Environ. Manag. 17, 118–121. doi: 10.1002/csr.236

[ref45] Patton M. Q., (2002). Qualitative research and evaluation methods. Thousand Oaks. Cal.: Sage Publications, 4.

[ref46] Pettigrew A. M. (1979). On studying organizational cultures. Adm. Sci. Q. 24, 570–581. doi: 10.2307/2392363

[ref47] Piwowar-Sulej K. (2020). Pro-environmental organizational culture: its essence and a concept for its operationalization. Sustain. For. 12:4197. doi: 10.3390/su12104197

[ref48] Schein E. H. (1986). Organizational culture and leadership. San Franciso CA: Jossey-Bass Publishers.

[ref9001] Schneider B. Karen M. B. (eds). (2014). The Oxford Handbook of Organizational Climate and Culture, Oxford Library of Psychology, online edn, Oxford Academic, 4 Aug. 2014, doi: 10.1093/oxfordhb/9780199860715.001.0001 (Accessed February 6, 2025).

[ref49] Tahir R. Athar M. R. Faisal F. Shahani N. un N. Solangi B.. (2019) ‘Green Organizational Culture: A Review of Literature and Future Research Agenda. Annals of Contemporary Developments in Management & HR (SSRN Scholarly Paper No. 3497251). Available at: https://papers.ssrn.com/abstract=3497251

[ref50] Tsang A. Frost T. Cao H. (2023). Environmental, social, and governance (ESG) disclosure: a literature review. Br. Account. Rev. 55:101149. doi: 10.1016/j.bar.2022.101149

